# Protocol for the mesh matters project: evaluating the efficacy of suture mesh vs. planar mesh in small ventral hernia repair – a randomized, blinded multicenter study

**DOI:** 10.1007/s10029-026-03815-3

**Published:** 2026-07-31

**Authors:** Vitaly Gameza, Nils Brandenburger, Peter Leutscher, Kathrine Holte

**Affiliations:** 1 Department of Gastrointestinal Surgery, North Denmark Regional Hospital, Bispensgade 37, 9800 Hjoerring, Denmark; 2 Centre for Clinical Research, North Denmark Regional Hospital, Hjoerring, Denmark; 3 Department of Clinical Medicine, Faculty of Medicine, Aalborg University, Aalborg, Denmark

**Keywords:** Ventral hernia, PROM, Surgical technique, Randomized trial, Polypropylene mesh

## Abstract

**Purpose:**

Small ventral hernia repair is among the most performed general surgical procedures. Despite the routine use of planar polypropylene mesh, surgical site occurrences (SSO) remain a frequent cause of morbidity, readmission, and patient dissatisfaction. A novel mesh-suture integrates mesh reinforcement with suture-like implantation and may reduce subcutaneous dissection and tissue trauma. The aim of this randomized trial is to investigate differences in SSO, patient-reported outcomes (PROMs) and operation time among patients operated for small ventral hernias either with this novel mesh-suture or planar mesh as well as evaluate potential influence of mesh-suture on wider socio-economic parameters such as return to work and contact with family physician/primary sector as well as long-term occurrence.

**Methods:**

A prospective, parallel-group, patient- and assessor-blinded, randomized controlled superiority trial conducted across Danish regional hospitals. Eligible patients undergoing elective repair of small ventral hernias are randomized 1:1 to mesh-suture or planar polypropylene mesh. The primary endpoint is SSO. Secondary endpoints include operative time, patient-reported quality of life (EQ-5D-5L, Abdominal Hernia Q), long-term recurrence, reoperation rates, and socio-economic outcomes. Analysis follows the intention-to-treat principle.

**Conclusion:**

This trial evaluates whether mesh-suture reduces wound morbidity while maintaining equivalent durability. Results may inform future standards in ventral hernia repair and device selection.

**Trial registration:**

The study is registered at clinicaltrials.gov om March 16th, 2026 (NCT07476560).

## Introduction

### Background and rationale

Surgery for small ventral hernias (< 2 cm) is one of the most frequently performed operations in Denmark, with 2469 procedures performed in 2023. Despite it being a relatively small operation, up to 7.1% of patients are readmitted to emergency departments < 90 days after surgery, primarily due to wound problems, and up to 2.2% of patients are reoperated < 30 days after surgery [1]. Historically, the two most important outcome measures following small ventral hernia repair are postoperative wound events and long-term hernia recurrence [2]. Current standard treatment of these hernias in Denmark is open surgery with onlay planar mesh placement. With an onlay planar mesh technique, the hernia sac is dissected and reduced, the abdominal wall defect is closed with nonabsorbable suture, the subcutaneous tissue is elevated from the underlying fascia, and a nonabsorbable mesh is placed over the fascia. This method is proven superior to closure of the hernia defect with only a suture in terms of recurrence rates [3, 4]. There is, however, a higher risk of seroma/hematoma formation and wound infection when planar mesh in the onlay position is used compared to suture closure [5, 6]. Rates of postoperative seroma formation are reported to be as high as 46% and in our clinical practice, readmission to hospital is predominantly caused by seroma formation related to the operative site [7].

Reported rates of surgical site occurrences (SSO) after operations for small ventral hernias vary greatly in the literature, additionally there is a lack of standardized definitions and reporting [2]. In settings comparable to ours, one study from Sweden showed complication rates of 23.7% after onlay planar mesh repairs [8]. The higher rate of hernia recurrence with suture only repair is attributed to the suture cutting through tissue, which occur when the sharp leading edge of the suture applies focused pressure at the suture/tissue interface and the pressure causes either abrupt cutting of tissue or a more gradual process of tissue ischemia and scarring, remodeling over time. In experimental studies, low suture tension has been found to promote more favorable collagen composition of the incisional region [9]. Planar mesh in the onlay position redistributes tension forces over a large surface area so the edges of fascia have optimal conditions for healing, but at the cost of higher wound complications rates and longer operation time.

#### Explanation for the choice of comparators

Several methods have been developed to reduce fascial tension during healing without extensive dissection of suprafascial or subfascial tissue. Among those are mesh sutured repairs where 2 cm wide mesh strips are used to close fascial defects just as with single tread interrupted suture. Mesh sutured repairs showed promising results even in contaminated fields and larger defects [10]. Mesh sutured technique is however subject to many variables that may alter its efficacy, such as accurate strip cutting, strip passing technique and mesh availability at the hospital.

Recently, a mesh-suture has been developed (our proposed study intervention), with an attached needle and a tubular design with pores in the wall, combining functions of both suture and mesh, allowing ingrowth of tissue without capsule formation, while having the tensile strength nearly identical to those of a 0-polypropylene suture [11]. Available data support the safety and efficacy of this mesh suture for abdominal wall closure [12]. By using mesh-suture, extensive subcutaneous dissection is avoided and surgical trauma is minimized, hypothesizing reduced wound complication rates.

### Objectives

The aim of this randomized, blinded multicenter trial is to investigate differences in SSO, patient-reported outcomes (PROMs) and operation time among patients operated for small ventral hernias either with mesh-suture or planar mesh. Furthermore we evaluate potential influence of mesh-suture on wider socio-economic parameters such return to work and contact with family physician/primary sector as well as long-term occurrence.

## Methods

**Fig. 1:**
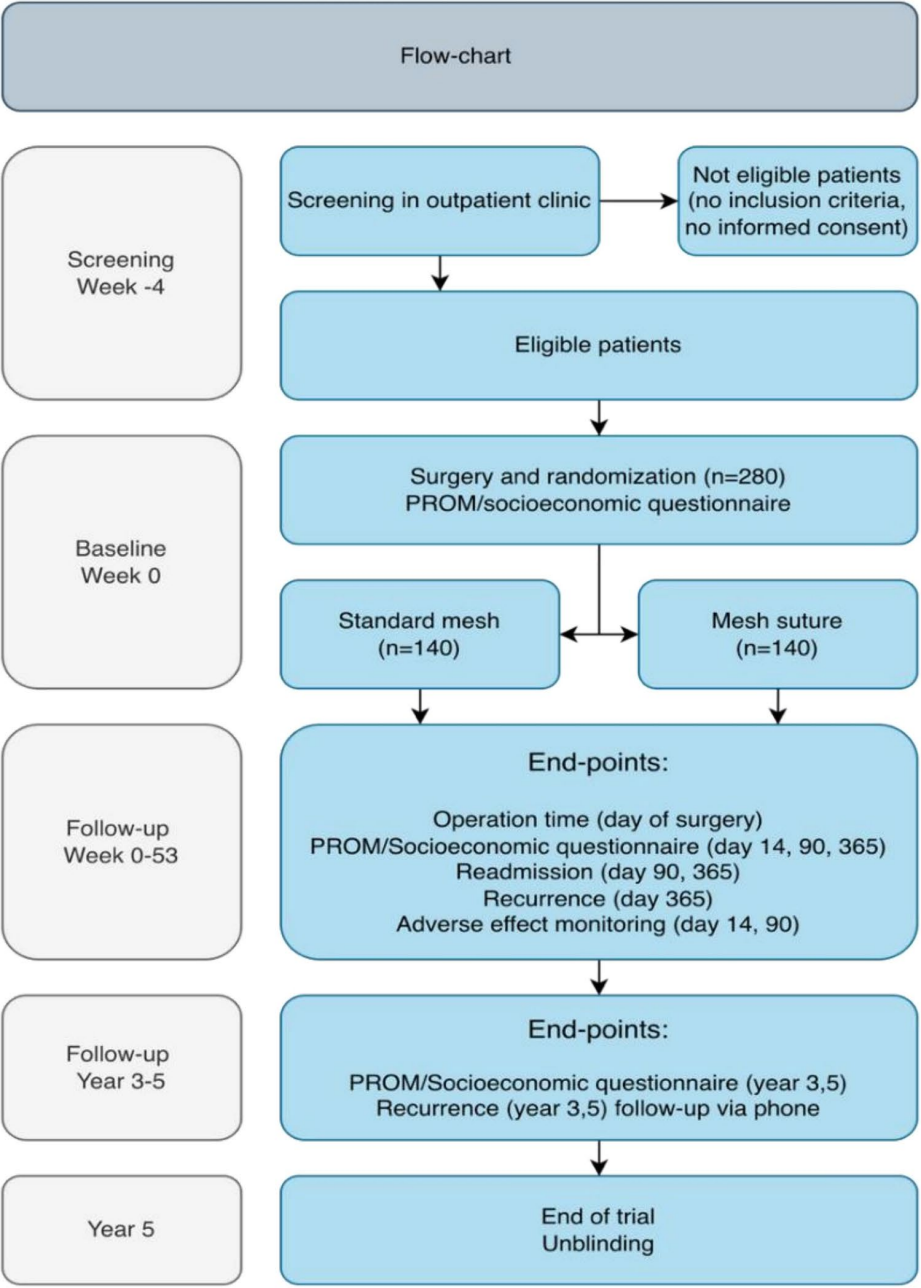
Flowchart for the study

The general study design and data flow are illustrated in Fig. 1. This protocol follows the SPIRIT 2025 guidelines [13]

### Trial design

Multicenter, randomized, patient- and outcome assessor-blinded trial with 1:1 allocation ratio. The study is powered for superiority with intention-to-treat analysis as primary analytic framework.

### Trial setting

Department of Gastrointestinal Surgery, North Denmark Regional Hospital, is the primary site, with additional participating centers in Denmark (names in progress).

### Eligibility criteria

Inclusion criteria:

- All patients presenting with small ventral hernia, maximum 3 × 3 cm, are evaluated for inclusion in the trial. Indication for open surgery with planar mesh in the onlay position is found, generally for hernia below 2*2 cm according to national guidelines. Hernias larger than 2*2 cm may, in exceptional circumstances, be found suitable for open surgery with planar mesh in the onlay position according to patient comorbidity or patient preference. This is at the discretion of the surgeon. The criteria for open surgery (smoking cessation 6 weeks prior to surgery, BMI < 35, diabetes treatment should be optimised to a glycated haemoglobin A1C (HbA1C) level of < 60 mmol/mol or 7.5%.) are described in the guidelines of the Danish Hernia Database [14] and all participating centres are obliged to follow them.

Exclusion criteria:

- Age < 18 years
- Acute operation
- Pregnancy
- Women with plans of future pregnancies
- Withdrawal of informed consent during admission

### Intervention and comparator

We compare two medical devices: Mesh-suture (intervention) vs. planar mesh (standard treatment).

In **the control group** we will use polypropylene mesh (Optilene® Mesh), a monofilament polypropylene mesh, pore size 1,5 mm, weight 60 g/m^2^. After implantation, the mesh adapts to the longitudinal and latitudinal expansions taking place in the connective tissue and is coloured blue with copper phthalocyanine (Phthalocyaninato (2-) copper) for a better visibility. The polypropylene mesh is biostable and is not degraded in the body.

In **the intervention group** we will use polypropylene mesh-suture (Duramesh™), combining the principles of a mesh repair with the placement precision of a suture. It is constructed from monofilament polypropylene and has an open walled, hollow core design that allows fibrovascular incorporation into the device during healing and distributes forces at the suture-tissue interface, while minimizing the amount of implanted foreign material and surgical complexity required for implantation. The polypropylene mesh-suture is biostable and not degraded in the body. Use of mesh-suture is technically simple and resembles standard suture use, thus all surgeons who perform ventral hernia repair would easily be able to use the technique.

**Fig. 2:**
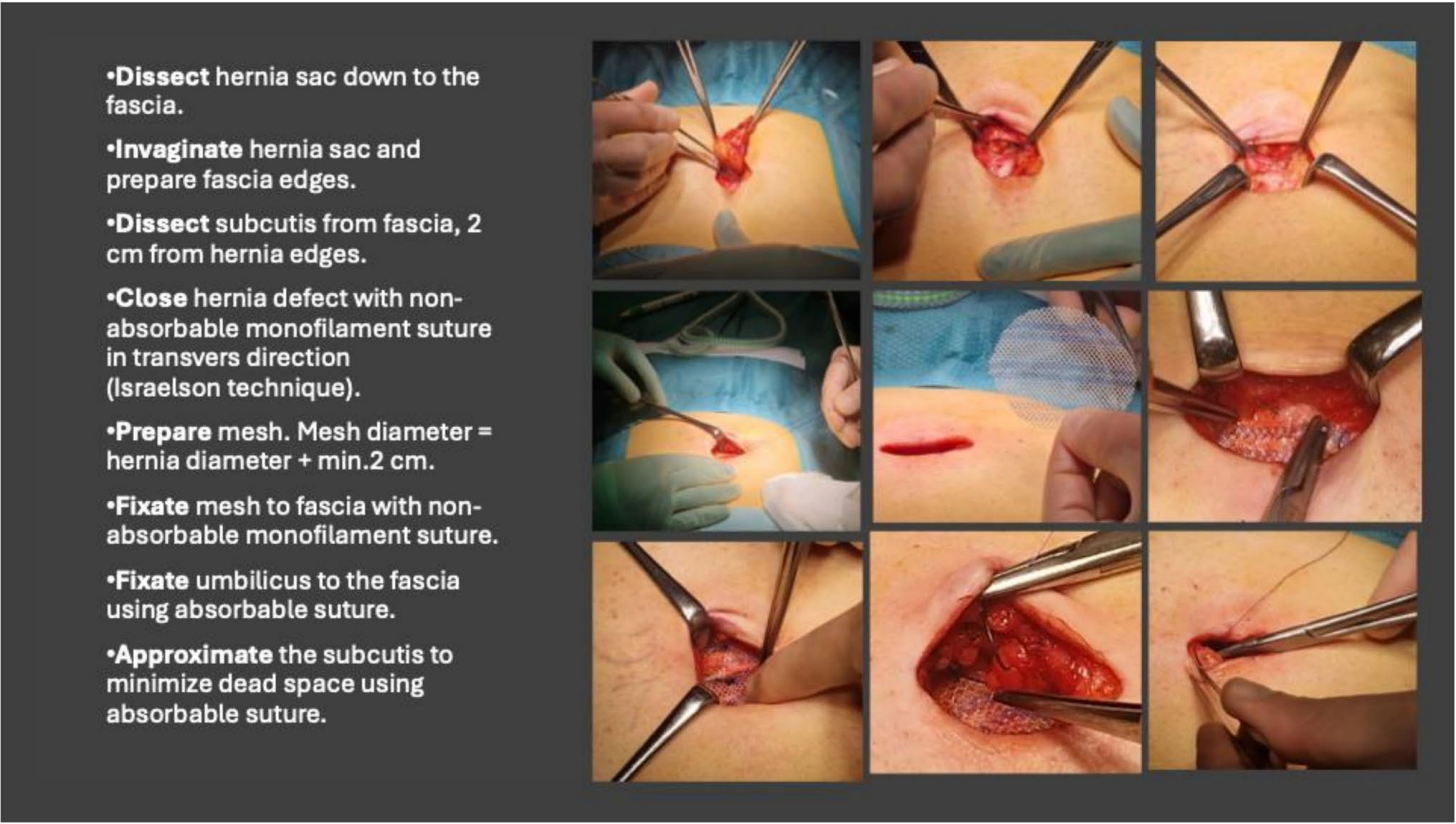

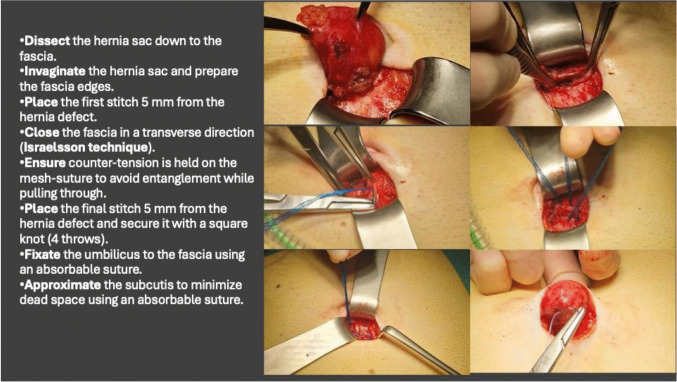
**a** Depiction of the surgical technique with planar mesh. **b** Depiction of the surgical technique with mesh-suture

Included patients will be randomized to either standard treatment with planar mesh in the onlay position or interventional treatment with mesh-suture. All hernia will be operated by consultant surgeons, fellows or residents after proven competency, possibly with supervision. Centers involved in the trial will be invited to a session where we go through operation details to secure that the procedure is performed in a standardized manner in all centers. Details of the operation with either planar mesh in the onlay position or mesh-suture are depicted in Fig. 2a and b.

#### Criteria for discontinuing or modifying allocated intervention

The main safety concern is a potentially higher recurrence rate among patients operated with mesh-suture. We will review all collected data after 3 months by a project member not otherwise involved in the project, and if early (< 90 days) recurrence rates in mesh suture subgroup exceed national average reoperation rates (2%), the study will be terminated.

#### Strategies to improve adherence to intervention

The study offers flexible scheduling visits, accommodating patients' work and personal commitments*.*

#### Concomitant care that is permitted or prohibited during the trial

Concomitant care is not restricted during this trial.

### Outcomes

#### Primary outcome

SSO on postoperative day 7–13. Definition: We apply the standardized CDC (Centers for Disease Control and Prevention) [15] and VHWG (Ventral Hernia Working Group) [2] definitions for SSO. Evaluation: Clinical examination. Additionally, an ultrasound examination will be performed. We have chosen to suture the skin with nonabsorbable suture. Normally this would be removed at a primary care visit at day 10. Instead, we see the patients in our outpatient clinic for our own evaluation including ultrasound. Thus the main argument for choosing this day is not to impose an extra visit on the patient as well as increase adherence to follow-up.

#### Secondary outcomes

1. PROMs on postoperative day 7–13, 90 and after 1 year. Definition and evaluation: EQ-5D-5L (generic) [16] and Abdominal Hernia-Q (hernia specific) [17] questionnaires.
2. Duration of surgery (min).
3. Long-term recurrence after 1, 3 and 5 years.
4. Socio-economic aspects (specifically time to return to work, analgesic use and contact with the primary sector) in both groups on postoperative day 7–13, 90 and after 1, 3 and 5 years.

### Participant timeline

**Fig. 3:**
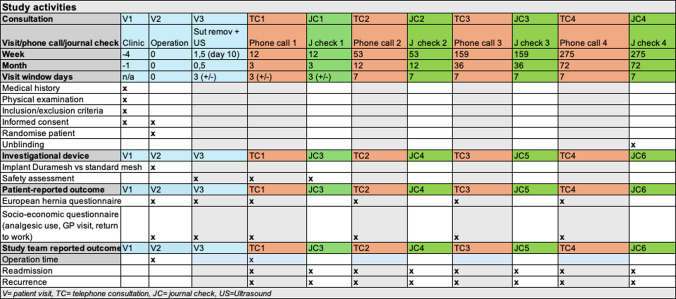
Study activity diagram. V = Visit. JC = Journal check. TC = Telephone call

Data collection will occur over 5 years from randomization of each patient and includes baseline, hospital visit (surgery) and follow-up. Details in Fig. 3.

### Sample size

In our department the readmission rate after small ventral hernia repair is 7% (data from the National Hernia Database, 2023) [1]. Most of these readmissions is due to wound complications. In the literature, wound complication incidences vary greatly (from 0.7 to 63.3%), at least in part due to a lack of standardized definitions and reporting [2]. We estimate that least as many patients are treated for minor SSO in primary sector as in the hospitals (unpublished data), so the true incidence of wound complications is estimated around 14 percent for onlay mesh. Based on these distinctions, our *realistic* expectation for wound complications is **1–3%.** However, to remain appropriately conservative, a reduction in wound complications from 14 to 4% is thus considered clinically significant.

A sample size of 254 participants was determined to have 80% power to detect a clinically significant between-group reduction in SSO from 14 to 4% and a type I error of 5%, without increase in recurrences. This number was increased to 280 participants to account for conversions and cases lost to follow-up.

### Recruitment

Patients referred to our outpatient hernia clinic, fulfilling the inclusion criteria, will be informed about the study and informed consent will be sought.

### Randomisation

#### Sequence generation

Patients will be randomized in a 1:1 ratio using a random sequence generator with random block size (of maximum 10).

#### Allocation concealment mechanism

The randomization table was generated by a person not involved in data collection and concealed from the study team.

#### Implementation

Patients will be enrolled during preoperative consultation in the outpatient hernia setting. After provision of consent, randomization is performed by the operating surgeon during the operation, the moment it is ensured that more/larger fascia defects than anticipated are not found. In all participating centers, a case of sequentially marked opaque envelopes will be made available containing patient study ID, information on the assigned intervention group and the registration schematics.

### Blinding

#### Blinding of trial participants

Blinding is maintained by ensuring that the operative report only states: *“… a mesh is implanted according to the study protocol and randomisation number …”*. This prevents patients from being able to see which type of mesh was used. Patients will be blinded until the end of follow-up, which is planned to be 5 years.

#### Blinding of the assessing physician

The physician who performs the follow-up assessment for wound complications is never the operating surgeon. Furthermore, the local database is configured so that the assessing physician does not have access to the section where the operating surgeon records the specific type of implanted mesh. All patients will be contacted by a study group member who is unaware of randomization and treatment given in order to collect PROM data.

### Data collection methods and management

#### Plans for assessment and collection of outcomes and data management

Study data are collected and managed using the encrypted REDCap electronic data capture tools hosted at Region Nordjylland. Patient data will be handled in accordance with “Databeskyttelsesloven og -forordningen”, the Danish adaptation of the European Union General Data Protection Regulation (GDPR). If patients are not interested in study participation, no data will be collected for the study.

Baseline data and preoperative QoL score will be collected during the first visit in the out-patient clinic or immediately preoperatively, if the patient consents to study participation. All patients will be called in for clinical examination on postoperative day 7–13, where standardized wound inspection and ultrasound will be performed just as QoL will be assessed by a standardized, validated questionnaire. QoL will be assessed by telephone on day 90, 1 year, 3 years and 5 years after surgery as well. Operation time is defined as time from first incision to last cutaneous suture and is registered by the floor-nurse in the operation room. 90 days after surgery patient journals will be assessed concerning data on potential readmissions.

#### Plans to promote participant retention and complete follow-up

All meetings are planned with the participants to accommodate their wishes best possible within the framework of the study. After the study period has ended, all personal information will be destroyed in line with the Danish and European guidelines for data management and protection. Throughout the trial, the conduction follows the Danish Data Protection Agency guidelines.

## Statistical methods

Data will be analyzed by a blinded analyst on an intention-to-treat basis, according to the prespecified statistical plan. A two-sided P-value < 0.05 is considered significant.

Analysis of the primary and secondary outcomes: Depending on the distribution of data, continuous variables will be described with mean (SD, standard deviation) or median [interquartile range (IQR)] and analyzed with students t-test or Mann–Whitney U test, respectively. Categorical variables will be described as frequencies (%) and analyzed using Fisher´s exact test. No interim analyses are planned.

### Oversight and monitoring

The coordinating investigator, a medical specialist and a dedicated hernia surgeon with more than 12 years of surgical experience, has the general responsibility for study monitoring and will secure that all legal and practical aspects of the study protocol are followed in all participating centers.

The coordinating investigator will receive weekly reports of performed operations from all participating centers and secure that all relevant data are submitted to a central database. If there is any baseline data missing, participants will be contacted. The database will be monitored on a weekly basis. Medical journals of all participants will be accessed on day 90 after the operation to secure all relevant data are collected. Access to data after submission will be restricted only to relevant study investigators to comply with the blinded study design. All study participants are contacted on day 90 after operation and if there is any suspicion of deviation from the expected postoperative course, participants will be called in for physical/radiological examinations. All study participants are informed to contact medical emergency services anytime if required. If recurrence appears, patients will be offered standard treatment with planar mesh in the onlay position.

Patients will expectedly be subject to increased awareness regardless of whether they are randomized to the intervention group or standard treatment, as this is a known phenomenon of study participation. This may make treatment safer in both groups.

## Ethics

### Research ethics approval

The study has been approved on October 28th 2025 by the Danish National Committee on Health Research Ethics (no. 2502170) and subsequently registered with the North Denmark Region (F2025-172, article 30).

### Protocol amendments

Protocol modifications will be communicated to relevant parties upon approval.

### Consent or assent

Written informed consent is obtained by hernia specialists during preoperative visit.

### Confidentiality

To ensure data security and participant confidentiality, all electronic records pertaining to the clinical study are maintained in password-protected databases, with access limited to approved study members. Written consent forms are stored in study binders in a locked office. When data sharing is required, such as for statistical analysis, only de-identified information is provided using secure file transfer protocols.

### Ancillary and post-trial care

Study participants will not receive financial compensation or other benefits and will be treated according to the Helsinki declaration. Complications directly attributable to the treatment will be eligible for insurance claims from the Danish public patient insurance (“Patienterstatningen”). Routine postsurgical care continues through normal clinical pathways.

## Open science

### Trial registration

The study is registered at clinicaltrials.gov om March 16th, 2026 (NCT07476560).

### Data sharing

Final dataset access is limited to study investigators and statisticians. Data sharing agreements are required for external access.

### Funding and conflicts of interest

The study is investigator initiated and sponsored by the surgical department of Regionhospital Nordjylland, Hjørring and the Clinical Research Unit, Regionhospital Nordjylland, Hjørring and external funding will be sought. None of the researchers have any economic affiliations to potential funds or other entities with economic interest in the study.

### Dissemination policy

The Declaration of Helsinki II will be adhered to and SPIRIT 2025 guidelines and checklist followed in reporting of the protocol [13]. CONSORT guidelines will be followed in reporting of the trial, and results will be published in international peer-reviewed journals, within 6 months of primary end-point completion, regardless of findings*.*

## Discussion

This randomized controlled trial is, to our knowledge, the first to evaluate mesh-suture vs. planar mesh in the onlay position in small ventral hernia.

One of the principles of good surgical practice is to cause as minimal surgical trauma as possible. By using mesh suture, we may substantially reduce the trauma caused by surgery by avoiding dissection of subcutaneous tissue from underlying fascia, preserving the perforating arteries and furthermore avoid creating an artificial space between subcutis and fascia where seroma and abscesses may accrue. By reducing surgical trauma, we hypothesize better QoL regarding, among others, pain, discomfort and mesh related complications such as foreign body sensations related to the surgical site. Reduced tissue trauma may facilitate faster recovery, earlier return to work, and decreased reliance on primary care services, potentially lowering the overall socioeconomic burden. Our expectations are to reduce operation time by 30%, which will potentially allow us to operate on more patients per day. Furthermore, we will assess hernia recurrence rates until 5 years postoperatively to ensure that mesh suture is not inferior to planar mesh in the onlay position. Hernia occurrence is an important surgical outcome, however increasing attention is drawn to the importance of patient-centered outcomes in surgical care [16]. We therefore use modified versions of the EuraHSQoL score and Abdominal Hernia-Q [17], validated for ventral hernia repair.

A systematic review of clinical studies suggested mesh-suture may be associated with low rates of incisional hernia, but the available evidence remains limited by study quality and high risk of bias, highlighting the need for randomized, controlled trials with long-term follow up before widespread implementation can be recommended [18]. To our knowledge, several randomized clinical trials are currently evaluating mesh suturing for trocar-site hernias; however, none have examined this approach in small ventral hernias [19].

### Strengths and limitations

The investigator-initiated, randomized design with patient and outcome assessor blinding as well as long-term outcome assessment is a major strength of this study. Conclusions on certain subgroups (ie patients with BMI > 35, smokers) not eligible for elective repair as per the Danish Hernia Database guidelines [14] may not be made as well as for patients undergoing emergency repair.

Planar mesh in the online position is the most common repair type chosen for these small hernia in Denmark [20]. As per the Danish National Hernia Database the recommendations for this surgery without risk factors is either preperitoneal or planar onlay mesh. The European and American Hernia Societies (EHS/AHS) recommend preperitoneal mesh placement for this hernia type [21]. The onlay planar mesh method is a technically simpler procedure, which is probably why it is more commonly performed in Denmark, for probably two main reasons: Most of these small hernias are operated in non-hernia centres by non-specialized hernia surgeons, furthermore these operations are largely used to train junior surgeons in the absolute beginning of their career. Thus, it is an important limitation of this study that we will not be able to make conclusions on mesh-suture in international settings where EHS/AHS guidelines are followed and preperitoneal mesh placement used. Accordingly, the primary clinical relevance of this trial lies within healthcare systems where onlay mesh repair remains the standard approach, particularly in Denmark. The applicability of the findings to countries and specialized abdominal wall centers routinely performing preperitoneal mesh repair should therefore be interpreted with appropriate caution.

In the future it will be important to compare Duramesh with preperitoneal mesh placements to increase international validity.

## Conclusions and clinical implications

The results of this trial are expected to be primarily relevant to healthcare systems where onlay mesh repair remains the standard treatment for small ventral hernias, particularly in Denmark. Further comparative studies against preperitoneal mesh repair will be required before the role of mesh-suture can be established in settings following current EHS/AHS recommendations.

### Trial status

Recruitment began January 5th 2026 and is anticipated to be completed within 1.5–2 years depending on the number of participating centers.

## References

[1] Dansk Herniedatabase Annual Report 2023. https://www.herniedatabasen.dk/_files/ugd/02befe_537096ef6bb64c56afa93214cbb0e77d.pdf. Accessed July 1st, 2026

[2] Haskins IN, Horne CM, Krpata DM et al (2018) A call for standardization of wound events reporting following ventral hernia repair. Hernia 22:729–736. 10.1007/s10029-018-1748-629429064 10.1007/s10029-018-1748-6

[3] Dias Rasador AC, da Silveira CAB, Lima DL et al (2024) Mesh versus suture for elective primary umbilical hernia open repair: a systematic review and meta-analysis. Hernia 28:2069–207839001938 10.1007/s10029-024-03106-9

[4] Henriksen NA, Jensen KK, Bisgaard T, Helgstrand F (2022) Suture or mesh repair of the smallest umbilical hernias: a nationwide database study. World J Surg 46. 10.1007/s00268-022-06520-110.1007/s00268-022-06520-135306587

[5] Kaufmann R, Halm JA, Eker HH et al (2018) Mesh versus suture repair of umbilical hernia in adults: a randomised, double-blind, controlled, multicentre trial. Lancet 391. 10.1016/S0140-6736(18)30298-810.1016/S0140-6736(18)30298-829459021

[6] den Hartog D, Dur AHM, Tulnebreijer WE, Kreis RW (2008) Open surgical procedures for incisional hernias. Cochrane Database Syst Rev. 10.1002/14651858.CD006438.pub1010.1002/14651858.CD006438.pub2PMC892495118646155

[7] Bisgaard T, Kehlet H, Bay-Nielsen M et al (2011) A nationwide study on readmission, morbidity, and mortality after umbilical and epigastric hernia repair. Hernia. 10.1007/s10029-011-0823-z21538150 10.1007/s10029-011-0823-z

[8] Bergström M, Widhe B, Granåsen G et al (2024) Onlay mesh versus suture repair for smaller umbilical hernias in adults-early results from SUMMER trial: randomized clinical trial. BJS Open 9. 10.1093/BJSOPEN/ZRAE17310.1093/bjsopen/zrae173PMC1187932540037347

[9] Höer JJ, Junge K, Schachtrupp A et al (2002) Influence of laparotomy closure technique on collagen synthesis in the incisional region. Hernia. 10.1007/s10029-002-0070-412209295 10.1007/s10029-002-0070-4

[10] Lanier ST, Dumanian GA, Jordan SW et al (2016) Mesh sutured repairs of abdominal wall defects. Plast Reconstr Surg Glob Open 4. 10.1097/GOX.000000000000106010.1097/GOX.0000000000001060PMC505502727757361

[11] Dumanian GA, Tulaimat A, Dumanian ZP (2015) Experimental study of the characteristics of a novel mesh suture. Br J Surg 102. 10.1002/bjs.985310.1002/bjs.9853PMC475839626154703

[12] Hackenberger PN, Mittal M, Fronza J, Shapiro M (2023) Duramesh registry study: short-term outcomes using mesh suture for abdominal wall closure. Front Surg 10:1321146. 10.3389/fsurg.2023.132114638274351 10.3389/fsurg.2023.1321146PMC10809794

[13] Chan AW, Boutron I, Hopewell S et al (2025) SPIRIT 2025 statement: updated guideline for protocols of randomised trials. BMJ 389. 10.1136/bmj-2024-08147710.1136/bmj-2024-081477PMC1203567040294953

[14] Danish Hernia Database (2025) Danish hernia database ventral Hernia algorithm 2025. https://www.herniedatabasen.dk/_files/ugd/02befe_b4ec550ff77b40d89fc79af87729963a.pdf. Accessed July 1st, 2026

[15] Surgical Site Infection Event (CDC). Surgical site infection (SSI) prevention guideline | infection control | CDC. https://www.cdc.gov/nhsn/pdfs/pscmanual/9pscssicurrent.pdf 10.1089/109629600321173 12594900

[16] Rosenberg J, Gram-Hanssen A, Reistrup H, Baker JJ (2025) Redefining success in hernia surgery: the case for patient-reported outcomes. J Clin Med 14. 10.3390/jcm1417613110.3390/jcm14176131PMC1242979840943891

[17] Sørensen CB, Gram-Hanssen A, Rosenberg J, Baker JJ (2025) Danish validation of the abdominal Hernia-Q. Dan Med J. 10.61409/A0624043440302704 10.61409/A06240434

[18] Nip L, Zhao S, Thomas R et al (2025) Mesh suture and mesh strips to prevent incisional hernia following abdominal wall closure or ventral hernia repair: systematic review. J Abdom Wall Surg 4:1457340443741 10.3389/jaws.2025.14573PMC12120353

[19] Nip L, Zhao S, Thomas R et al (2025) Duramesh™ versus conventional suture for prevention of trocar-site hernia following laparoscopic surgery (TROCAR): study protocol for a double-blind randomised controlled trial. Trials. 10.1186/s13063-025-09388-341466434 10.1186/s13063-025-09388-3PMC12829174

[20] Witthøft C, Ahmed U, Rosenberg J, Baker JJ (2026) Mesh placement and risk of reoperation for recurrence after incisional hernia repair: a nationwide register-based cohort study. Hernia 30. 10.1007/s10029-026-03690-y10.1007/s10029-026-03690-yPMC1320128742184040

[21] Henriksen NA, Montgomery A, Kaufmann R et al (2020) Guidelines for treatment of umbilical and epigastric hernias from the European Hernia Society and Americas Hernia Society. Br J Surg 107:171–19031916607 10.1002/bjs.11489

